# Supplementary material for: Are carbon emissions trading and green financial instruments synergistic? -Comprehensive quantitative research based on content analysis
Source: PLoS One. 2024 Mar 7;19(3):e0298601. doi: 10.1371/journal.pone.0298601 (PMC10919601; doi:10.1371/journal.pone.0298601)
Supplement: S1 Data — (ZIP) [file pone.0298601.s001.zip › Data/地级市Z_F工作报告.zip!/╔╜╢1⁄2/╢1⁄2╙¬╩╨/└┤╫╘╣1⁄2╓┌║┼í╛┬φ┐╦╩2╛▌═°í┐_2014.docx]

各位代表：

　　现在，我代表市人民政府向大会报告政府工作，请予审议，并请市政协委员和其他列席会议的同志提出意见。

　　一、2013年工作回顾

　　过去的一年，在市委的正确领导下，全市认真贯彻落实中央、省委省政府决策部署及市委工作会议精神，牢牢把握“高境界、高标准、高起点、大作为”的工作总要求，紧紧围绕“转方式、调结构、扩总量、增实力、上水平”的工作大局，务实创新，积极作为，经济社会发展取得显著成绩。

　　(一)积极应对困难挑战，经济保持平稳较快发展。在外需萎缩、经济下行的困难情况下，我们及时采取一系列针对性措施，保持经济平稳较快发展，主要指标增幅居全省前列。初步统计，2013年全市生产总值达到3250亿元，同比增长11%。规模以上工业总产值达到1.17万亿元，增长19.5%，主营业务收入过百亿企业达到24家。实现公共财政预算收入183.79亿元，增长15.8%。全社会固定资产投资达到2300亿元，增长21.5%。社会消费品零售总额达到584亿元，增长13.2%。金融机构各项存款余额2848亿元、贷款余额2161.3亿元，分别比年初增加453.2亿元、349.9亿元。

　　(二)加快调整产业结构，转型升级取得新进展。积极发展高效生态农业，实施了总投资268亿元的22个重点项目，渔业、畜牧、食用菌等优势产业规模效益不断提升。农业龙头企业发展到593家，农民专业合作社发展到1339家。加强农业基础设施建设，完成河道治理、灌区改造等一批水利工程，建设高标准农田8.7万亩。加快推进新型工业化，围绕传统产业技术改造、先进制造业和战略性新兴产业发展实施了529个项目，高新技术产业比重达到33.4%。认真落实推进服务业跨越发展若干政策，金融、物流等重点产业快速发展，服务业增加值增长9.6%。强化科技创新支撑，实施省级以上科技项目114项，国家采油装备工程技术研究中心、黄河三角洲现代农业国际创新园获批组建，国瓷公司获得我市首个中国专利金奖。全国质量强市示范城市创建工作深入推进。

　　(三)加强城市和基础设施建设，城乡面貌发生显著变化。中心城实施了52项重点工程。大剧院、体育馆等九大公益场馆完成主体；金湖银河水系开挖和湿地建设基本完成，园博园主展馆开工建设。东城工业品市场、西城商河路商业街等片区改造基本完成，建设改造城区道路19公里。加快城市绿化美化，建设改造城市公园7处，新增绿地115万平方米。积极推进县城和小城镇建设，新建改造农村社区38个、农村公路500公里，黄河南展区村庄改造扎实推进。创建中国人居环境奖通过验收，城乡环境综合整治取得明显成效。加强重大基础设施建设。东营港十大业主码头和公共管廊带建成投用，东营至旅顺滚装航线复航。胜利机场新开通四条航线。德大铁路建设扎实推进，黄大铁路、东营港疏港铁路获国家、省核准。济东高速东营段、广利港疏港公路开工建设。大唐东营电厂前期工作进展顺利。

　　(四)狠抓环境保护和生态建设，环境质量逐步改善。加强水气污染治理，实施环保重点工程76项，新建城乡污水处理厂10座，取缔土小污染企业182家。大力推进节能减排，淘汰落后产能项目37个，实施节能技术改造项目116个、重点减排工程项目61个、循环经济项目58个，万元生产总值能耗、污染物排放量等实现控制目标。继续加强造林绿化，完成造林12.4万亩。自然保护区修复湿地10万亩，被列入国际重要湿地名录。

　 (五)加大统筹发展力度，区域一体化发展步伐加快。油地团结共建不断拓展，签署了油地共建协同发展框架协议，完善了联席会议制度，一批共建项目顺利推进，展现出油地大融合、大发展的广阔前景。油地校合作共建中国石油大学胜利学院启动实施。军地协商解决了一批事关区域发展的重大问题。市属两个开发区产业聚集明显加快；各县区比学赶超、争先进位，县域经济实力进一步增强。

　　(六)深化改革开放，发展活力不断增强。深入推进重点领域改革。出台了加快金融改革发展的意见，民间融资规范引导试点取得阶段性成果，市县地方金融监管机构挂牌运转。“营改增”试点稳步推进。农村综合改革取得明显成效。实施未利用地开发14.2万亩，净增耕地5.8万亩。精简行政审批事项102项。公共资源交易中心挂牌运营。食品药品、工商、质监管理体制改革顺利推进。扩大对外开放，实现进出口总值128亿美元，其中出口56亿美元，分别增长3.5%和12.4%；实际利用外资1.9亿美元，增长19.1%；境外投资增长52%，对外工程营业额3.3亿美元。

　　(七)繁荣发展社会事业，社会保持和谐稳定。坚持教育优先发展，深入实施中小学办学条件标准化等工程，新建改造学校、幼儿园116所，高中段学校布局调整和小班化教育试点启动实施。加快发展卫生事业，市立儿童医院建成投用；公立医院全部实行药品零差率销售，县级及以下公办医疗机构全面实行“先诊疗后结算”服务模式；城镇居民医保与新农合率先并轨运行。繁荣发展文化事业，成功承办十艺节优秀剧目展演，文化惠民工程深入实施。创建全国文明城市取得较好成绩。加强精神文明和民主法制建设，“六五”普法工作扎实有效。我市运动员在第十二届全运会上取得历史最好成绩。加强人口计生工作，持续稳定低生育水平。加强平安东营建设，开展社会治安环境集中整治百日行动，认真做好信访工作，社会保持和谐稳定。国防教育和双拥共建深入开展，军政军民团结更加巩固。对口支援取得新进展。

　　(八)持续保障改善民生，人民群众得到更多实惠。城镇居民人均可支配收入33890元、增长9.5%，农民人均纯收入12640元、增长10%。十件民生实事完成年度计划。有效促进重点群体就业，新增城镇就业4.61万人。新型农民学校培训41万人次，农村劳动力转移就业4.72万人。社会保障水平进一步提高，城乡居民养老、医保、低保、五保等保障标准居全省前列。建设保障性住房1.5万套，油田职工住房新一轮工程启动。新建改造城乡养老设施115处，市级养老产业园开工建设。社会福利、慈善和残疾人事业快速发展。对中心城6处农贸市场升级改造，环境面貌显著改善。

　　各位代表，人民群众的生命财产安全是最大的民生。我们以创建国家安全发展示范城市为抓手，进一步落实企业主体责任和政府部门监管责任，深入开展安全生产大检查、“平安行·你我他”活动和“打非治违”专项行动，加强油气安全综合整治，采取明查暗访的方式，督促整改了一批安全隐患，安全生产形势保持稳定。

　　各位代表，过去的一年，以市委工作会议召开为标志，拉开了东营新一轮大发展大跨越的序幕。实践证明，解放思想是推动干事创业的总开关，必须不断提升思想境界和工作标准，用解放思想指导实践，提升境界谋划发展，创新理念推进工作。推动新一轮发展是全市上下的根本任务，必须加强战略谋划，创新思路举措，集中解决一批事关全局和长远发展的根本问题。项目建设是实现跨越发展的“牛鼻子”，必须紧紧抓在手上，靠项目做强产业、靠项目转型升级、靠项目调整结构、靠项目增强实力、靠项目支撑发展。提质增效是转方式调结构的中心，必须贯穿于经济发展的各方面各环节，落实到推动工作的全过程，体现到政策措施的落实中，实现更高层次更高质量发展。保障改善民生是经济社会发展的根本目的，必须坚持以人为本，加大民生投入，每年实施一批民生实事，不断增进群众福祉。作风建设是推动事业发展的重要保障，必须大力弘扬求真务实的优良作风，着力推进工作落实，营造干事创业的浓厚氛围。这些成功经验，既是全市人民在推动新一轮发展实践中的深刻体会，也是指导东营未来发展的重要启示，必须长期坚持，不断丰富发展。

　　一年来成就的取得，是在中央、省委省政府和市委正确领导下，全市上下、油地军校、社会各界团结奋斗、携手共建的结果。在此，我代表市政府，向辛勤劳动的广大干部群众，向积极支持政府工作的人大代表、政协委员和社会各界人士，向所有关心、帮助东营发展的同志们、朋友们，表示崇高的敬意和衷心的感谢！

　　同时，我们也清醒地看到，我市发展中还存在一些问题。受国际国内大环境影响，生产总值、进出口等指标没能达到预期目标，保持经济平稳较快发展的压力依然较大；经济结构性矛盾突出，重化工业比重过大，服务业发展滞后，新兴产业尚未形成规模优势；农业抗御自然灾害能力薄弱；发展方式仍然粗放，自主创新能力不强；发展的动力活力不足，对外开放的层次和水平不高；资源环境制约不断加剧，环境保护和节能减排压力巨大；安全生产隐患较多；社会公共服务与群众期盼还有差距；机关作风、服务效能需要进一步提升。对此，我们一定要高度重视，采取有效措施，认真加以改进。

二、面临的形势和2014年工作总体要求

　　2014年，是贯彻落实党的十八届三中全会精神、全面深化改革的第一年，是推动东营新一轮发展的攻坚年。做好今年的政府工作，意义重大而深远。

　　综观国内外形势，世界经济仍将延续缓慢复苏态势，我国发展仍处于可以大有作为的重要战略机遇期，具备经济持续健康发展的基础条件。随着十八届三中全会一系列政策的落实，必将释放强大的改革红利。经过建市三十年的发展，我市综合实力大幅提升，内生动力显著增强，黄蓝国家战略深入实施，“投资洼地”效应更加凸显。尤其是去年召开的市委工作会议，为东营今后的发展指明了方向、凝聚了力量。同时也要看到，国内外发展环境十分复杂，不稳定不确定因素仍然较多。我们要切实增强机遇意识、发展意识、改革意识，既要充分看到有利条件和积极因素，又要充分估计困难和挑战，改革创新，积极作为，奋力开创经济社会发展的新局面。

　　今年政府工作的总体要求是：以党的十八届三中全会和习近平总书记系列重要讲话精神为指导，坚持稳中求进工作总基调，以全面深化改革为统领，以“转作风、促发展，提标准、上水平”为主题，深入实施黄蓝国家战略，更加注重优化经济结构，更加注重推进改革开放，更加注重强化创新驱动，更加注重生态环境建设，更加注重保障改善民生，保持经济持续健康发展和社会和谐稳定，努力推动东营大发展大跨越。

　　今年全市经济社会发展的预期目标是：生产总值增长11%左右，全社会固定资产投资增长18%，社会消费品零售总额增长13%，进出口总值、吸收外商直接投资保持适度增长，公共财政预算收入增长11%左右，城镇居民人均可支配收入增长11%左右，农民人均纯收入增长12%左右，城镇登记失业率控制在2.4%以内，居民消费价格涨幅控制在3.5%左右，人口自然增长率控制在8.07‰以内，全面完成省政府下达的各项节能减排约束性目标。

　　实现上述目标，要坚持“三高一大”的工作总要求不动摇，坚持“转方式、调结构、扩总量、增实力、上水平”的工作大局不动摇，坚持“一个龙头、三个增长极”的发展布局不动摇，坚持推进工作转变、提升发展质量效益不动摇，坚持推进改革创新增创发展新优势不动摇，坚持统筹兼顾促进全面发展不动摇，着力推动东营新一轮发展上水平。只要全市上下、油地军校、方方面面万众一心，携手共进，集聚正能量，再鼓新干劲，就一定能克服一切艰难险阻，夺取经济社会发展的新胜利！

三、2014年重点工作

　　(一)在抓好牵动全局的大事上取得更大突破。做好今年的工作，必须坚持以事关长远、牵动全局的大事为抓手，扎实开展“三大专项行动”、全力抓好“十件大事”，带动整体工作上水平。

　　三大专项行动：一是城市公共安全提升年行动。以中心城为重点，突出油气管线安全、道路交通、建设工程、人员密集场所等重点领域，全面排查整治安全隐患；强化安全生产责任落实，持续开展安全生产大检查，严厉打击非法违法行为，确保全市不发生重特大安全生产责任事故，确保不造成重特大人员伤亡和经济损失，确保不因安全生产责任事故影响干扰全市新一轮发展。二是生态环境整治提升年行动。铁腕治理水气污染，严厉惩治破坏生态环境和危害群众健康的环境违法行为。坚决淘汰落后产能，严把新上项目能评环评安评关口，实施一批重点减排和治理工程，加强生态建设，促进环境质量全面改善。三是乡村文明建设提升年行动。按照“整体启动、分类推进，突出重点、标本兼治，制定规范、全面提升”的原则，统筹推进生态文明乡村建设、乡村文明行动，推进村庄净化、硬化、绿化、亮化、美化和城乡环卫一体化，改善群众生产生活条件，提升农村文明程度。

　　十件大事：第一，加快东营港建设，开工建设北防波堤、2个突堤及沿线泊位，启动实施10万吨深水码头建设；抓好已建成码头和滚装航线运营，拓展散杂货和集装箱业务，扩大港口吞吐能力。第二，依托仙河镇规划建设东营港城，完善提升规划，启动起步区基础设施建设，兴港公路建成通车。第三，力争开工建设东营港疏港铁路。第四，启动广利港建设，加快疏港公路建设。第五，实施市一中、二中、中专学校迁建，优化学校布局，提升办学水平。第六，加快建设市人民医院急诊急救中心暨内科综合病房楼，提升医疗服务水平。第七，实施“137”计划和“双十工程”，培育一批“台柱子”企业。第八，推进设立东营综合保税区，打造高层次开放平台。第九，支持东营农业高新技术产业示范区创建国家级农高区。第十，深入开展全国文明城市创建工作，确保顺利通过考核验收。

　　(二)在加快经济转调上迈出更大步伐。推动经济转型升级，是东营新一轮发展的主攻方向和目标要求。要以提高质量效益为中心，扩大产业规模，提升产业层次，着力打造东营经济升级版。

　　抓大项目好项目促转调。坚持把项目建设作为加快转调的主要抓手，加快推进294个亿元以上续建项目，开工建设310个亿元以上项目，突出抓好70个市级重点建设项目。认真研究国家产业政策，精心筛选论证项目，做到开工建设一批、洽谈签约一批、论证储备一批。完善项目推进机制，推进签约项目快落地、落地项目快开工、开工项目快投产、投产项目快达效。注重项目建设质量和效益，突出抓好高新技术产业、现代服务业、节能环保和现代农业项目。

　　加快新型工业化促转调。一是推动传统产业向高端提升。围绕石油化工、有色金属、橡胶轮胎等优势产业，实施以新装备、新技术、新工艺为核心的改造工程，重点抓好198个过亿元项目，促进存量优化、工艺升级、产品升档。二是推动先进制造业高质高效发展。围绕石油装备、汽车及零部件等产业，坚持高端化、集约化、品牌化方向，重点抓好93个过亿元项目，提升自主设计、制造和成套能力，提高研发、生产和经营管理水平。三是推动战略性新兴产业加快突破。围绕高端装备制造、新材料、新能源、新一代信息技术、新医药等产业，重点抓好73个过亿元项目，带动新兴产业跨越发展。鼓励传统工业企业向战略性新兴产业转型发展。四是推动信息化和工业化深度融合。支持发展物联网、电子商务和信息产业，集中抓一批“两化”融合发展示范企业、示范项目，提升企业自动化、智能化、管理现代化水平。

　　突破发展现代服务业促转调。认真落实政策措施，集聚各类资源向服务业倾斜，促进服务业突破发展。一是加快发展金融服务业。规划建设金融集聚区，重点引进外资银行、金融机构地区总部、功能中心，加快构建多层次金融服务体系。加快企业上市步伐，推动华东石油交易中心等要素市场建设发展。支持保险业深度发展。二是加快发展现代物流业。围绕石油化工、橡胶轮胎等主导产业，加快建设一批产业关联度高、特色鲜明的现代物流园区，引进培育一批大型物流企业，推动物流业集聚发展。三是加快发展文化旅游业。突出抓好重点景区总体规划和产品开发，积极发展乡村旅游，提升旅游吸引力和综合效益。四是加快发展服务外包业。加强公共平台建设，实施软件园三期工程，引进一批行业领军企业，提高服务外包业规模和水平。鼓励引导企业剥离非主营业务，促进生产性服务业加快发展。把发展服务业与促进消费结合起来，扩大消费需求，推动消费升级。

　　支持企业发展促转调。企业是市场的主体，也是转调的主体。必须从政策上倾斜，从要素资源上支持，从排忧解难上服务，形成支持服务企业发展的良好氛围。稳定骨干企业生产，提升企业运行质量。继续实施市级领导联系重点企业和驻企联络员制度，帮助企业解决融资、土地、市场等方面的困难和问题。积极扶持中小微企业发展，鼓励融入大企业产业链，推动快速成长。加强企业家队伍建设，着力培养一批高素质的新生代企业家。

　　增强科技创新能力促转调。深入实施创新驱动战略。建设一批创新园区，重点推动可持续发展研究院、大学科技园、胜利科技创新园等园区建设。提升一批创新平台，重点抓好工程技术研究中心、企业重点实验室等建设，增强产业自主创新能力。壮大一批科技型企业，组织实施重大科技项目，引导更多企业成为创新主体。完善一批科技创新服务机构，加强国家科技创新服务体系试点城市建设，推进科技金融融合，激发创新发展活力。深入实施人才强市战略。以高层次和高技能人才为重点，统筹推进“黄河三角洲学者”、“金蓝领”培训等重点人才工程，强化创业人才政策扶持，加强知识产权运用和保护，推动多出人才、多出精品。

　　(三)在提升“三农”工作水平上下更大气力。“三农”问题事关“两个率先”的大局，必须按照“三个导向”的要求，着力转变农业发展方式，推进农业现代化，促进农民持续增收。

　　加快农业结构调整。大力发展高端高质高效种植养殖业，继续深化粮棉菜高产创建活动，鼓励发展种子、种苗研发培育等产业，提升农业增量。围绕畜牧、渔业、食用菌、林果、蔬菜、花卉和休闲观光农业等优势产业，创新种养模式，推广应用农业新技术、新品种、新工艺，优化农业存量。加大先进适用农机具示范推广力度，加快农业机械化进程。

　　大力发展园区农业。鼓励引导工商资本投向农业，发展不同层次、不同规模的农业园区，提高农业规模效益，加快建设国家现代农业示范区。支持现代畜牧业示范区加快发展。加强家庭农场建设，年内发展到200家。

　　提升农业产业化水平。大力发展农产品加工业，实施一批精深加工项目，促进农产品转化增值。加大农业龙头企业扶持力度，力争年销售收入过10亿元企业发展到12家。更加注重品牌策划和宣传营销，打造区域性物流交易平台，提高黄河口名优农产品市场占有率。整合检测资源，加大监管力度，确保农产品质量安全。培育农民专业合作组织等新型生产经营主体，组织农民进入产业链经营。

　　着力改善农村生产生活条件。大搞农田水利基本建设，利用3-5年时间，从根本上改善农业生产条件。今年重点实施55个项目，改善除涝面积170.6万亩、灌溉面积69.3万亩。加快实施广饶、利津村级公路网化示范工程，新建改造农村公路480公里，搞好农村电网续建配套。加快推进南水北调配套工程和重点园区水源工程，完善黄河口中心渔港基础设施。

　　(四)在促进区域协调发展上争取更大作为。按照区域互动、优势互补、共同发展的原则，充分发挥各方优势，优化资源要素配置，加快构筑区域协调发展新格局。

　　着力推进油地军校协同共建。油地军校团结结合，是东营发展的独特优势和强大动力。我们要牢固树立“一家人、一盘棋”的思想，高举团结发展的旗帜，完善合作机制，提升合作水平，携手推动区域发展繁荣。深入推进工作结合，支持胜利油田建设高产稳产大油田，支持石油大学提高教学科研水平，支持济军生产基地加快改革发展。深入推进资源整合，挖掘区域资金、科技、人才、市场、土地等资源潜力，规划建设特色产业园区等合作载体，引导油军校企业入园发展，培育新的经济增长点。深入推进力量融合，加快城市建设、民生保障、基础设施一体化步伐，进一步提高市民生活水平，携手共建和谐幸福美好家园。

　　着力打造各具特色的经济板块。按照“一个龙头、三个增长极”的发展布局，鼓励支持各县区、开发区在转型升级、改革开放、科技创新等方面率先突破，引领全市新一轮发展上水平。广饶县，要统筹推进工业化、信息化、城镇化、农业现代化，打造带动区域发展的大龙头、好龙头、强龙头。东营经济技术开发区，加快建设高新技术产业和先进制造业基地，打造转方式调结构的先行区、城市发展的新空间、全市新一轮发展的最大增长极。东营港经济开发区和河口区，坚持港区城一体发展，建设现代生态化工基地、国际物流港和现代化滨海城市，打造北部经济增长极。东营区和垦利县，着力发展石油装备、现代服务业和新兴产业，打造服务功能完善、高端要素聚集、辐射带动能力强的主城区增长极。利津县，要抓住全市支持发展的机遇，加快赶超步伐，整体提升发展水平。打破行政区域界限，促进东营港经济开发区、河口蓝色经济开发区、利津滨海新区一体化发展，推动北部沿海经济隆起带加快崛起。

　　(五)在推进新型城镇化上达到更高水平。推进以人为核心的城镇化，必须坚持尽力而为、量力而行，把握节奏、突出重点，着力提升城镇化质量和水平。

　　建设秀美宜居中心城市。高标准编制城市规划。突出东营因油而建、东城开发、新城建设不同时期历史文化特色，科学编制各类规划，严格规划管理，维护规划的严肃性和权威性。高水平推进城市建设。加快科技馆、青少年宫等续建项目建设进度；启动实施一批新建项目，城市道路重点抓好东一路南延等工程，生态建设重点抓好森林公园等工程，新区开发重点抓好生态新城、金湖银河等片区，旧城改造重点抓好油城广场等片区，市政公用设施重点抓好南郊水厂二期续建等项目。高效能加强城市管理。加快智慧城市建设，完善网格化管理模式，实现中心城区数字化管理全覆盖。以星级小区和社区品牌创建为抓手，推动实施便民服务进社区试点工程，全面提升小区规范化建设管理水平。按期完成园博园工程，全力办好第五届省园林绿化博览会。

　　推进城乡一体化发展。强化县域中心城市骨干支撑作用，推进县城建设。推动产城融合，全面加强小城镇规划建设，重点支持市级试点强镇、省级示范镇和国家级改革试点镇加快发展。坚持实事求是、因地制宜，稳妥推进新型农村社区建设，重点推进城中村、乡镇驻地村拆迁改造。加强城镇市政设施建设，完善提升社会公共服务功能，推进城镇基本公共服务常住人口全覆盖。建立完善城镇一体化管理体制机制，提高管理水平。

　　增强基础设施承载能力。提高胜利机场配套服务能力，加快国产大飞机试飞基地建设。推动黄大铁路、淄东铁路建设改造尽快开工，建设东营火车站，争取德大铁路、寿平铁路建成通车。加快济东高速东营段建设，做好长深高速、沿海高等级公路前期工作。加快推进大唐东营电厂、胜利电厂三期工程。积极推进沿海防潮堤建设。

　　(六)在加强生态文明建设上采取更实举措。加强生态文明建设是一项战略任务，加强环境保护是政府的一项重要职责，必须从污染治理入手，加快建设生态东营、美丽东营。

　　加强污染综合整治。全面实施重点流域水污染综合整治，完善企业污水处理设施，确保正常运行达标排放；完善生活污水处理设施及配套管网，加快建设东城南、西城北等城乡污水处理厂，确保达到“一镇一厂、水质改善”的目标；每条省控、市控河流至少建成一处湿地净化工程。大力实施大气污染综合治理工程，开展城区建设扬尘治理集中行动，绿化城市裸露土地，严控工业废气、烟尘和机动车尾气污染，加快淘汰黄标车并实施区域禁行，抓好燃煤电厂、城区燃煤锅炉和石化企业脱硫脱硝设施建设。加强空气质量监测，建设完善自动监测系统。加大环境执法力度，达不到排放标准要求的企业一律停产整治。

　　加强生态环境建设。加快造林绿化。完善林场经营管理体制，提高林木养护管理水平。继续抓好“三网”和重点绿化工程建设，实施沿海基干林带提升工程。继续实施金湖银河生态绿化工程，完善生态水系。持续推进自然保护区湿地修复，建设5处省级湿地公园。加强海洋特别保护区建设，争创国家级海洋生态文明示范区。

　　加强资源节约综合利用。实行能源消费强度和总量双控制，强化重点领域、重点行业、重点企业节能降耗。支持企业节能改造，深入开展能效对标活动，实行合同能源管理，严格清洁生产审核。推进建筑、交通、公共机构和居民生活等领域节能。全面落实最严格的水资源管理制度。研究出台土地利用管理办法，推动节约集约利用土地。加快推进循环经济园区和循环经济企业试点，大力推广循环经济关键链接技术和工艺。鼓励发展低碳经济。扩大风能、太阳能、地热等清洁能源的开发利用。

　　(七)在深化改革开放上实现更大进展。认真落实全面深化改革的各项措施，采取更加积极主动的开放战略，切实增强科学发展的动力与活力。

　　积极有序地深化改革。深化投资体制改革，确立企业投资主体地位。支持非公有制经济健康发展。做好未利用地开发利用试点、城乡建设用地增减挂钩试点。推进工商注册制度便利化。深化财税体制改革，培植高效财源，强化税收征管，保持财政收入稳定增长，提高财政收入质量；做好“营改增”试点工作；改进预算管理和监督，大力压减“三公”经费开支，集中财力保民生、保重点、保在建；健全政府性债务和资产管理制度。加强地方金融监管，鼓励金融创新。稳步推进农村综合改革，依法维护农民土地承包经营权，发展多种形式规模经营；鼓励农村发展合作经济，赋予农民更多财产权利；扎实做好各类农村产权的确权登记和颁证工作，探索建立农村产权流转交易市场。深化教育综合改革，全面实施素质教育，大力促进教育公平。深化医药卫生体制改革，统筹推进医疗保障、医疗服务、公共卫生、药品供应、监管体制综合改革。完善文化管理体制，扎实推进省级公共文化服务体系示范区创建。深化行政体制改革，积极稳妥推进事业单位分类改革，推广政府购买服务。加快生态文明制度建设，建立资源有偿使用和生态补偿制度，完善生态环境保护管理机制。

　　进一步扩大对外开放。实施市场多元化战略，推动出口稳定增长。转变外贸发展方式，加强重点产品出口基地建设和出口龙头企业扶持，促进加工贸易转型升级。鼓励企业扩大先进技术设备、关键零部件和能源原材料大宗商品进口。整合口岸监管资源，推动电子口岸建设。加强招商选资，瞄准国内外大企业，引进一批传统产业“提质增效”项目、高端服务业项目和高新技术项目。抓好中美清洁能源合作产业园建设，推动东营港新建码头对外开放，构筑一批对外开放的新平台。扩大对外投资，支持有条件的企业全球布局产业链，搞好境外资源合作开发、优势产能转移，拓展经济发展空间。

　　(八)在为群众谋福祉上取得更多实效。把维护人民群众利益作为全部工作的出发点和落脚点，扎实办好民生实事，加强和创新社会管理，让发展成果更多、更公平惠及全体人民。

　　在稳定和扩大就业方面：完善并落实就业创业扶持政策，新开发公益性岗位1000个以上，扶持创业5000人以上，带动就业2万人以上，创建一批创业型示范乡镇和示范社区。统筹抓好高校毕业生等重点群体就业，加快“一中心四园区”等创业基地建设。办好新型农民学校，增强群众创业致富能力。

　　在社会保障体系建设方面：积极推动社会保险由“制度全覆盖”到“人群全覆盖”转变，逐步提高基本养老保险、失业保险标准。城市低保标准每人每月提高30元，农村低保标准每人每年提高400元，农村五保供养标准每人每年提高800元。城乡居民医疗保险财政补助标准每人每年提高到360元，全面铺开医疗救助“一站式”即时结算。完善养老服务体系，建设6处养老机构、30处城市社区日间照料中心、70处农村幸福院、3处养老服务信息化平台。

　　在支持教育优先发展方面：全面实施数字化校园工程，加快中心城学校、幼儿园建设，启动第二轮学前教育三年行动计划。深入开展小班化教育试点，引导学校特色发展。支持东营职业学院建设国家骨干高职院校，加快市技师学院二期工程建设。推进油地校合作共建中国石油大学胜利学院，实现以新的办学体制面向全国招生。

　　在提高人民群众健康水平方面：继续推进公立医院改革，建立科学的医疗绩效评价机制和人才培养、人事薪酬制度。完善农村三级医疗卫生服务网络和城市社区卫生服务体系，加强基层医疗人才队伍建设，提高基本公共卫生服务经费标准。健全完善重大疾病防控机制，提升疾控机构实验能力。以稳定低生育水平为中心，强化基层基础，积极应对新形势，统筹解决人口问题，促进人口长期均衡发展。

　　在丰富群众精神文化生活方面：加强社会主义核心价值体系教育，深入实施“四德”工程，提高全社会文明程度。加强文化设施建设，实施“进千村乐万家”活动。加强网络管理，规范传播秩序。办好黄河口国际马拉松赛等活动，备战第二十三届省运会，加快建设10分钟健身圈。

　　在住房保障方面：实施保障房建设和棚户区改造工程，年内新建保障性住房1.13万套。完善保障性住房准入、退出和服务管理机制。建设61个新型农村社区，加快推进黄河南展区旧村拆迁改造工程。支持胜利油田、济军生产基地职工住房建设。

　　在食品药品安全方面：改革完善食品药品监管体制，有效整合检验检测资源，加强基层监管执法力量，逐步构建食品药品安全社会共治格局。加强食品药品安全过程控制，建立最严格的覆盖全过程的监管制度。建立食品原产地可追溯制度和质量标识制度。深入开展专项整治，从严查处食品药品安全违法违规行为。

　　在创新社会治理方面：改进社会治理方式，普及法律知识，运用法治思维和法治方式化解社会矛盾。以网格化管理、社会化服务为方向，健全基层综合服务管理平台。加强和改进信访工作，健全及时就地解决群众合理诉求机制。完善重大决策社会稳定风险评估和突发事件应急管理机制。深入推进平安东营建设，完善社会治安防控体系，依法严密防范和惩治各类违法犯罪活动，严厉打击欺行霸市、强揽工程、阻挠施工等不法行为。

四、加强政府自身建设

　　做好今年的政府工作，必须认真开展以为民务实清廉为主要内容的群众路线教育实践活动，坚决贯彻市委转变作风真抓实干的要求，不断增强抓推进、抓落实、抓成效的能力，切实担负起推动新一轮发展的重任。

　　一要解放思想、提升境界。在新的起点上推动新一轮发展，必须破除自我感觉良好的自满心态、破除传统的思维方式、破除只重当前不谋长远的短视观念，不断强化忧患意识、争先意识、机遇意识，以更高境界更高标准谋划和推进新发展新跨越。坚定不移地推进改革创新，用新的体制机制解决问题，用新的方式方法推动工作。始终保持奋发有为的精神状态，以矢志不移的决心、扭住不放的韧劲、攻坚克难的勇气，把东营的事业奋力推向前进。

　　二要改进作风、扎实苦干。认真落实中央八项规定，坚决反对“四风”。深入开展发展环境集中整治行动，继续实施“效能提升”工程，加强“庸懒散”专项治理。大力弘扬求真务实优良作风，讲实话、干实事，敢作为、勇担当，言必信、行必果。精简会议文件，改进会风文风，把更多精力放到研究工作、谋划发展上来。倡导扎实深入的工作作风，接地气、听民声、察民情、解民忧。强化工作目标责任制，严格督查考核，严明工作纪律，确保政令畅通、令行禁止，不断增强政府公信力和执行力。

　　三要依法行政、科学决策。严格按照法定权限、法定程序行使权力、履行职责、管理事务，切实做到守规矩、重程序、按制度办事。健全重大问题集体决策、专家咨询、跟踪问效等机制，提高政府决策的民主化、科学化水平。深化行政审批制度改革，进一步简政放权。完善行政执法程序，确保严格规范公正文明执法。自觉接受人大法律监督、工作监督和政协民主监督，扎实做好人大代表建议和政协提案办理工作，广泛接受社会各方面监督。

　　四要廉洁勤政、执政为民。严格落实党风廉政建设责任制，扎实推进惩治和预防腐败体系建设，建立完善长效机制。强化重要岗位、关键环节的监督制约，让人民监督权力，让权力在阳光下运行，把权力关进制度的笼子。坚决纠正损害群众利益的不正之风，严肃追究失职渎职行为，严厉查处各种违纪违法案件。严格执行党政机关厉行节约反对浪费条例和公务接待规定，建设节约型机关。全体政府工作人员要严格执行廉洁从政各项规定，努力做到干部清正、政府清廉、政治清明。

　　各位代表，推动新一轮发展，使命光荣、责任重大。让我们在市委的坚强领导下，勇于担当，锐意进取，以百倍的信心、百倍的努力、百倍的干劲，为加快推进“两个率先”、推动东营新一轮大发展大跨越而努力奋斗！
